# A_2A _and A_3 _adenosine receptor expression in rheumatoid arthritis: upregulation, inverse correlation with disease activity score and suppression of inflammatory cytokine and metalloproteinase release

**DOI:** 10.1186/ar3527

**Published:** 2011-12-06

**Authors:** Katia Varani, Melissa Padovan, Fabrizio Vincenzi, Martina Targa, Francesco Trotta, Marcello Govoni, Pier Andrea Borea

**Affiliations:** 1 Department of Clinical and Experimental Medicine, Pharmacology Unit, University of Ferrara via Fossato di Mortara 17-19, 44121 Ferrara, Italy; 2 Department of Clinical and Experimental Medicine, Rheumatology Section, University of Ferrara via Fossato di Mortara 17-19, 44121 Ferrara, Italy

## Abstract

**Introduction:**

The reduction of the inflammatory status represents one of the most important targets in rheumatoid arthritis (RA). A central role of A_2A _and A_3 _adenosine receptors (ARs) in mechanisms of inflammation has been reported in different pathologies. The primary aim of this study was to investigate the A_2A _and A_3_ARs and their involvement in RA progression measured by Disease Activity Score in 28 or 44 joints (DAS28 or DAS).

**Methods:**

ARs were analyzed by saturation binding assays, mRNA and Western blotting analysis in lymphocytes from early and established RA patients. The effect of A_2A _and A_3_AR agonists in nuclear factor kB (NF-kB) pathway was evaluated. Tumor necrosis factor-α (TNF-α), interleukin-1β (IL-1β) and interleukin-6 (IL-6) release was carried out by A_2A _and A_3_AR activation. AR pharmacological regulation in matrix metalloproteinase-1 (MMP-1) and metalloproteinase-3 (MMP-3) release was also studied.

**Results:**

In lymphocytes obtained from RA patients, A_2A _and A_3_ARs were up-regulated if compared with healthy controls. A_2A _and A_3_AR activation inhibited the NF-kB pathway and diminished inflammatory cytokines such as TNF-α, IL-1β and IL-6. A_2A _and A_3_AR agonists mediated a reduction of MMP-1 and MMP-3 release. A_2A _and A_3_AR density inversely correlated with DAS28 and DAS suggesting a direct role of the endogenous activation of these receptors in the control of RA joint inflammation.

**Conclusions:**

Taken together these data demonstrate that the inflammatory and clinical responses in RA are regulated by A_2A _and A_3_ARs and support the use of A_2A _and/or A_3_AR agonists as novel and effective pharmacological treatment in RA patients.

## Introduction

Rheumatoid arthritis (RA) is a chronic inflammatory disease characterized by progressive joint destruction associated with synovial proliferation and secretion of high levels of pro-inflammatory mediators, including cytokines and growth factors [1,2]. Early diagnosis and therapy are crucial in order to prevent unfavorable outcome with joint deterioration and functional disability [3]. Therapeutic strategies are primarily based on disease modifying anti-rheumatic drugs (DMARDs) alone or in combination with biological drugs, in case of inadequate response. This represents the most innovative and effective treatment to slow the progression of the disease [4,5]. To date, rheumatologists have widely adopted the 28-joint Disease Activity Score (DAS28) or 44-joint Disease Activity Score (DAS) as validated and reliable composite indexes to assess RA disease activity [6-8].

In RA the inflammatory process leads to the advancing and permanent degradation of the cartilage with synovial hyperplasia, change in underlying bone and high levels of inflammatory mediators [9,10]. It is widely accepted that cytokines such as tumor necrosis factor-α (TNF-α), the interleukin-1 (IL-1) family and interleukin-6 (IL-6) have various important activities in the context of the RA pathogenesis [11]. In particular, IL-6 interacts in complex ways with the cells involved in bone remodelling indirectly, promoting osteoclastogenesis and contributing to the severity of the radiological joint damage [12]. Many proteinases are expressed in joint tissues of RA patients even if among them different matrix metalloproteinases (MMPs) are believed to have a key role in joint destruction [13]. Of these, MMP-1 and MMP-3 are particularly important because they are produced by fibroblast-like synoviocytes and monocytes/macrophages in the synovium and are known to play a key role in tissue destruction [14,15].

Several papers report a central role of adenosine receptors (ARs) in mechanisms of inflammation associated to various pathologies suggesting that their stimulation has a different effect on the release of several pro-inflammatory cytokines [16-18]. Adenosine, a purine nucleoside, is considered a potent regulator acting with four cell surface receptor subtypes - A_1_, A_2A_, A_2B _and A_3_ARs, which are coupled to different G proteins [19,20]. The A_1 _and A_3_ARs exert an inhibitory effect on cAMP production while A_2A _and A_2B_ARs mediate an increase of cAMP accumulation [21-24]. In RA patients, adenosine suppresses the elevated levels of pro-inflammatory cytokines such as TNF-α and IL-1β [25,26]. It has also been shown that A_3_ARs are over-expressed in patients with autoimmune inflammatory diseases, including RA, and that A_3_AR pharmacological treatment modulates an improvement in signs and symptoms [27-29].

From this background it is accepted that the release of various inflammatory mediators in RA patients could be closely associated with ARs, suggesting their potential role as therapeutic target and the application of novel pharmacological approaches in the treatment of RA. Additional data regarding a possible correlation between validated disease activity scores, such as DAS28 or DAS, and AR density could be very important in better ascertaining the modulation of joint inflammatory status and, as a consequence, the disease progression.

In a previous study, our group showed that A_2A _and A_3 _ARs are up-regulated in early RA (ERA) patients and after methotrexate treatment but not in RA patients treated with anti-TNF-α agents [30]. To confirm these data in a larger cohort of patients, we have investigated in this study the presence of A_1_, A_2A_, A_2B _and A_3_ARs by saturation binding assays, mRNA and Western blotting analysis in human lymphocytes from RA patients with established disease more than 12 months and ERA patients with symptom duration less than 12 months in comparison with age-matched healthy subjects. The effect of A_2A _and A_3_AR agonists or antagonists in nuclear factor kB (NF-kB) activation and in the production of proinflammatory cytokines was evaluated. The joint degradation process that is believed to be largely mediated by proteases has been analyzed by evaluating the effect of A_2A _and A_3_AR agonists on MMP-1 and MMP-3 production. To shed some light on the role of ARs in the therapeutic approach, the correlation between A_2A _and A_3_AR density expressed as Bmax values with DAS28 and DAS was explored.

## Materials and methods

### Patients and control subjects

All patients enrolled in this study were recruited from the Rheumatology Section, Department of Clinical and Experimental Medicine, University of Ferrara, Italy. A total of 95 patients was included and divided into ERA patients (*n *= 32) and RA patients (*n *= 63). ERA patients were diagnosed according to the following criteria: symptom duration less than 12 months, synovitis of at least three joints, morning stiffness occurring of more than 30 minutes duration and exclusion of other common causes of arthritis. RA patients with established disease of more than 12 months fulfilled the American College of Rheumatology (ACR) 1987 criteria for rheumatoid arthritis [31]. All of the ERA patients were followed up for an additional 12-month period after which the ACR criteria were met. The demographic, clinical and pharmacological details are listed in Table 1.

**Table 1:** Clinical features and pharmacological treatments in RA patients

	Control subjects (*n *= 90)	ERA patients (*n *= 32)	RA patients (*n *= 63)
**Clinical parameters**			
N° of women/N° of men	53/37	28/4	55/8
Age, mean ± SEM years	55.2 ± 6.3	59.2 ± 2.5	62.7 ± 1.3
Duration of RA (mean ± SEM months)	-	5.1 ± 0.6	136.7 ± 15.2
Rheumatoid factor positive, RF, N°(%)	-	15 (46.9%)	42 (66.7%)
Anti-CCP antibody positive, N°(%)	-	14 (43.8%)	43 (68.3%)
DAS28 (mean ± SEM)	-	4.85 ± 0.25	4.92 ± 0.18
DAS (mean ± SEM)	-	3.44 ± 0.19	3.47 ± 0.14
HAQ (mean ± SEM)	-	1.21 ± 0.10	1.32 ± 0.12

**Pharmacological treatments**		**n (%)**	**n (%)**
			
NSAIDs	-	7 (21.9%)	5 (7.9%)
GC	-	19 (59.4%)	27 (42.9%)
GC + DMARDs	-	6 (18.8%)	31 (49.2%)
CyA	-	1 (3.1%)	0
HCL	-	2 (6.3%)	2 (3.2%)
MTX	-	3 (9.4%)	19 (30.2%)
LFN	-	0	10 (15.9%)

Anti-CCP, anti-cyclic citrullinated peptide; CyA, Cyclosporin A; DAS, Disease Activity Score; DMARDs, disease-modifying antirheumatic drugs; ERA, early rheumatoid arthritis; GC, glucocorticoids low dose, alone; HAQ, healthy assessment questionnaire; HCL, hydroxichloroquine. LFN, leflunomide; MTX, methotrexate; NSAIDs, nonsteroidal anti-inflammatory drugs; RA, rheumatoid arthritis

Disease activity was assessed using the validated indexes DAS28 and DAS, the Health Assessment Questionnaire (HAQ) was used to evaluate functional impairment, the anti-cyclic citrullinated peptide (anti-CCP) and rheumatoid factor (RF) were also checked by using a second generation ELISA method and nephelometry, respectively [32-34].

Healthy controls (*n *= 90), matched for similar age to the cohort of RA patients, were volunteers from Ferrara University Hospital Blood Bank. The study was approved by the local Ethics Committee of the University Hospital of Ferrara and informed consent was obtained from each participant in accordance with the principles outlined in the Declaration of Helsinki.

Additional data describing materials and detailed methods, including human lymphocyte preparation, real time quantitative polymerase chain reaction (RT-PCR), Western blotting, saturation binding experiments and ELISA are published in the online supplementary material.

### Sample collection and human lymphocyte preparation

Lymphocytes were isolated and prepared as previously described from the peripheral blood of control subjects, ERA and RA patients [30]. The isolation of blood cells started no later than three to four hours after the samples had been taken. The blood was supplemented with 6% (by weight) Dextran T500 solution (Sigma, St Louis, MO, USA) and erythrocytes were allowed to settle down for 60 minutes. Leukocytes were pelleted by centrifugation for 15 minutes at 100 g and the remaining erythrocytes were lyzed in distilled water at 4°C. Cells were pelleted by centrifugation for five minutes at 250 g, suspended in Krebs-Ringer phosphate buffer and layered onto 10 ml of Fycoll-Hypaque (GE Healthcare, Little Chalfont, UK). After centrifugation, mononuclear cells were washed in 0.02 M phosphate-buffered saline at pH 7.2 containing 5 mM MgCl_2 _and 0.15 mM CaCl_2_. Finally, they were decanted into a culture flask and placed in a humidified incubator (5% CO_2_) for 2 h at 37°C. This procedure, aimed at removing monocytes, which adhere to the culture flasks, resulted in a purified lymphocyte preparation containing at least 99% small lymphocytes identified by morphological criteria.

To obtain membrane suspensions, cell fractions were centrifuged in hypothonic buffer at 20,000 g for 10 minutes. The resulting pellet was resuspended in tris HCl 50 mM buffer pH 7.4 containing 2 UI/ml adenosine deaminase (Sigma) and incubated for 30 minutes at 37°C. After the incubation the suspension was centrifuged again at 40,000 g for 10 minutes and the final pellet was used for radioligand binding assays. The protein concentration was determined by a Bio-Rad method with bovine albumine as reference standard [30].

### Real-Time quantitative polymerase chain reaction (RT-PCR) experiments

Total cytoplasmic RNA was extracted by the acid guanidinium thiocyanate phenol method. Quantitative RT-PCR assays [22] of A_1_, A_2A_, A_2B _and A_3_AR mRNAs were carried out using gene-specific fluorescently labelled TaqMan MGB probe (minor groove binder) in a ABI Prism 7700 Sequence Detection System (Applied Biosystems, Warrington, Cheshire, UK). For the RT-PCR of A_1_, A_2A_, A_2B _and A_3_ARs the Assays-on-Demand™ Gene expression Products NM 000674, NM 000675, NM 000676 and NM 000677 were used respectively. For the RT-PCR of the reference gene, the endogenous control human β-actin kits were used, and the probe was fluorescent-labeled with VIC™ (Applied Biosystems, Monza, Italy).

### Western blotting analysis

Human lymphocytes were washed with ice-cold phosphate buffer saline containing 1 mM sodium orthovanadate, 104 mM 4-(2-aminoethyl)-benzenesulfonyl fluoride, 0.08 mM aprotinin, 2 mM leupeptin, 4 mM bestatin, 1.5 mM pepstatin A, 1.4 mM E-64 (Sigma). Then cells were lysed in Triton lysis buffer and the protein concentration was determined using BCA protein assay kit (Pierce, Rockford, IL, USA).

Aliquots of total protein sample (50 μg) were analyzed using antibodies specific for human A_1_, A_2B_, A_2A _and A_3_ARs (1 μg/ml dilution, Alpha Diagnostic, San Antonio, TX, USA) [22]. Filters were washed and incubated for one hour at room temperature with peroxidase-conjugated secondary antibodies (1:2,000 dilution). Specific reactions were revealed with Enhanced Chemiluminescence Western blotting detection reagent (GE Healthcare, Little Chalfont, UK).

### Saturation binding experiments to A_1_, A_2A_, A_2B _and A_3_ARs

Saturation binding experiments to A_1_ARs were performed by using (^3^H)-DPCPX ((^3^H)-1,3-dipropyl-8-cyclopentyl-xanthine, specific activity 120 Ci/mmol, Perkin Elmer Life and Analytical Sciences, Boston, MA, USA) as radioligand [30]. Human lymphocyte membranes (60 μg of protein/assay) with 8 to 10 concentrations of (^3^H)-DPCPX (0.01-20 nM) were incubated for 90 minutes at 25°C. Non-specific binding was determined in the presence of DPCPX 1 μM.

Saturation binding to A_2A_ARs was carried out with the use of (^3^H)-ZM 241385 ((^3^H)-4-(2-(7-amino-2-(2-furyl)(1,2,4)-*triazolo*(2,3-a)(1,3,5)triazin-5-ylamino)ethyl) phenol, specific activity 27 Ci/mmol, Biotrend, Cologne, Germany), as radioligand [30]. Cell membranes (60 μg of protein/assay) were incubated for 60 minutes at 4°C with various concentrations (0.01 to 20 nM) of (^3^H)-ZM 241385. Non specific binding was determined in the presence of ZM 241385 1 μM.

Saturation binding experiments to A_2B_ARs were analyzed using (^3^H)-MRE 2029F20 ((^3^H)-N-benzo(1,3)dioxol-5-yl-2-(5-(2,6-dioxo-1,3-dipropyl-2,3,6,7-tetrahydro-1*H*-purin-8-yl)-1-methy-1*H*-pyrazol-3-yl-oxy)-acetamide, specific activity 123 Ci/mmol, GE Healthcare, Little Chalfont, UK) as radioligand [30]. Cell membranes (80 μg of protein/assay) and (^3^H)-MRE 2029F20 (0.01 to 30 nM) were incubated for 60 minutes at 4°C and non-specific binding was determined in the presence of MRE 2029F20 1 μM.

Saturation binding experiments to A_3_ARs were assessed using [^3^H]-MRE 3008F20 ((^3^H)-5N-(4-methoxyphenylcarbamoyl) amino-8-propyl-2-(2-furyl) pyrazolo (4,3-e)-1,2,4-triazolo (1,5-c)pyrimidine, specific activity 67 Ci/mmol, GE Healthcare, UK) as radioligand [30]. The membranes (80 μg of protein/assay) with (^3^H)-MRE 3008F20 (0.01 to 30 nM) were incubated at 4°C for 150 minutes and MRE 3008F20 1 μM was used to evaluate non specific binding.

Bound and free radioactivity was separated by filtering the assay mixture through Whatman GF/B glass fibre filters by using a Brandel cell harvester. The filter bound radioactivity was counted in a 2810 TR liquid scintillation counter Packard (Perkin Elmer, Boston, MA, USA).

### Lymphocyte cell culture

Isolated lymphocytes from controls and RA patients were suspended at a density of 10^6 ^cells/ml in RPMI 1640 medium supplemented with 2% fetal bovine serum (Euroclone, Milan, Italy) and seeded into 24-well plates (1 ml/well). Cells were allowed to rest for two hours in a 37°C incubator in 5% CO_2_/95% air.

Cells were then pre-incubated for 15 minutes with 100 nM of CGS 21680 (4-(2-((6-Amino-9-(*N*-ethyl-β-D-ribofuran uronamidosyl)-9*H*-purin-2-yl)amino)ethyl)benzene propanoic, Sigma) or Cl-IB-MECA (N^6^-(3-iodo-benzyl)-2-chloro-adenosine-5'-*N*-methyluronamide, Sigma) in the absence and in the presence of selected A_2A _or A_3_AR antagonists. The antagonists used were SCH 442416 (2-(2-Furanyl)-7-(3-(4-methoxyphenyl)propyl)-7*H*-pyrazolo(4,3-*e*)(1,2,4) triazolo(1,5-*c*)pyrimidin-5-amine, Tocris, Bristol, UK) or MRS 1334 (1,4-Dihydro-2-methyl-6-phenyl-4-(phenylethynyl)-3,5-pyridinedicarboxylic acid 3-ethyl-5-((3-nitrophenyl)methyl) ester, Tocris) at 1 μM concentration, respectively. Adenosine agonists and/or antagonists were incubated before cell activation with 5 ng/ml phorbol myristate acetate (PMA) for 24 hours [35]. At the end of incubation the cell suspension was collected and centrifuged at 1,000 g for 10 minutes at 4°C. Then, the supernatants or cell pellets were used for ELISA assays or nuclear extract preparation, respectively.

### NF-kB activation in human cultured lymphocytes

Nuclear extracts from human cultured lymphocytes of the examined patients were obtained by using a nuclear extract kit (Active Motif, Carlsbad, CA, USA) according to the manufacturer's instructions. The NF-kB activation was evaluated by detecting phosphorylated p65 proteins in nuclear extracts by using the TransAM NF-kB kit (Active Motif). Phosphorylated NF-kB subunits specifically bind to the immobilized oligonucleotides containing the NF-kB consensus site (5'-GGGACTTTCC-3'). The primary antibody used to detect NF-kB recognized an epitope in the subunits that is accessible only when it is activated and bound to its DNA target. A horseradish peroxidase (HRP)-conjugated secondary antibody provided a sensitive colorimetric readout that was quantified by spectrophotometry at 450 nm wavelength [30].

### Pro-inflammatory cytokines release in cultured lymphocytes

TNF-α levels were measured in human cultured lymphocytes after the treatment described above by using highly sensitive TNF-α enzyme linked immunosorbent assay (R&D Systems, Minneapolis, MN, USA) according to the manufacturer's instructions.

Pro-inflammatory cytokine (IL-1β and IL-6) levels were determined with a quantitative sandwich ELISA kit (R&D Systems) according to the manufacturer's instructions [36]. The reaction was developed with streptavidin-horseradish peroxidase and optical density was read at 450 nm wavelength.

### Measurement of total MMP-1 and MMP-3 release in cultured monocytes

To obtain human monocytes, peripheral blood mononuclear cells were seeded in petri dishes at the density of 10^6^/ml. The cells were allowed to adhere to plastic tissues and non adherent cells (lymphocytes) were removed.

In cultured monocytes, MMP levels were measured after the treatment described above by using the corresponding quantitative sandwich ELISA kit (R&D Systems,) according to the manufacturer's instructions [37]. Briefly, the assay systems measure natural and recombinant human active and pro-MMPs (total MMPs).

### Statistical analysis

Dissociation equilibrium constants for saturation binding, affinity or K_D _values, as well as the maximum densities of specific binding sites, Bmax were calculated for a system of one or two-binding site populations by non-linear curve fitting using the program Ligand purchased from Kell Biosoft, Ferguson, MO, USA. All data are reported as mean ± SEM of different independent experiments as indicated in the Results section or in the Figure legends. Analysis of data was performed by one-way analysis of variance (ANOVA). Differences between the groups were analyzed with Dunnett's test and were considered significant at a value of *P *< 0.01. A simple regression model was used to analyze the linear dependence of clinical variables on B_max _values of A_2A _and A_3_ARs.

## Results

### A_2A _and A_3_ARs are up-regulated in RA patients

**Figure 1:**
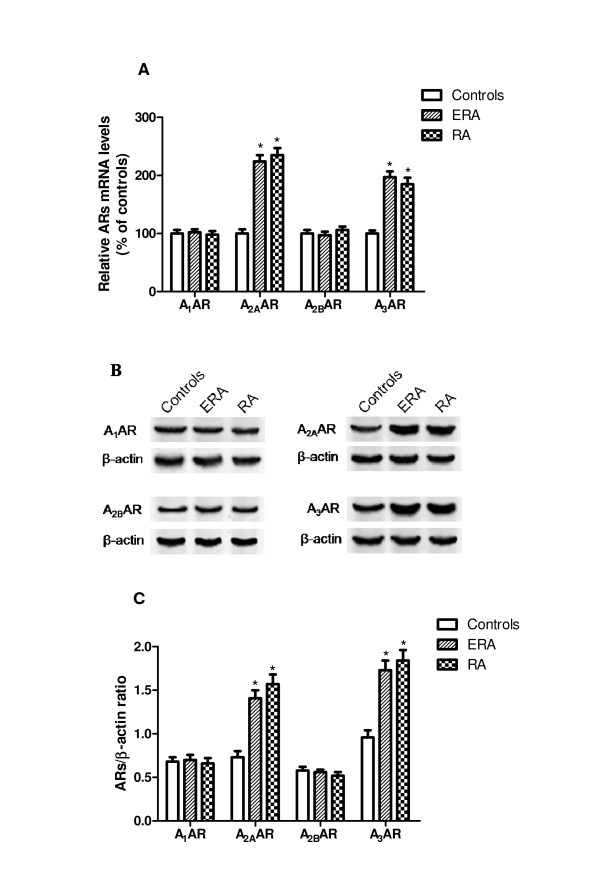
**A_1 _A_2A _A_2B _and A_3_ARs in RA patients**. **A) **Relative AR mRNA levels determined by RT-PCR in human lymphocytes from ERA (*n *= 32), RA patients (*n *= 63) and control subjects (*n *= 90). **B) **Representative Western blotting analysis shows immunoblot signals of ARs in ERA, RA patients and controls. **C) **Densitometric analysis of AR expression in human lymphocytes from ERA (*n *= 32), RA patients (*n *= 63) and control subjects (*n *= 90) indicated as a ratio of β-actin (loading control). Data are expressed as a means ± SEM. *, *P *< 0.01 vs control group.

Figure 1A reports the relative A_1_, A_2A_, A_2B _and A_3_AR mRNA levels determined by RT-PCR in human lymphocytes from healthy subjects, and ERA and RA patients. Among these receptors only A_2A _and A_3_AR mRNA expression in RA patients was significantly increased. Western blotting and densitometric analysis in lymphocytes indicate a significant increase in A_2A _and A_3_AR protein expression in RA patients compared to healthy subjects while no differences were found in A_1 _and A_2B_ARs (Figure 1B, C).

**Figure 2:**
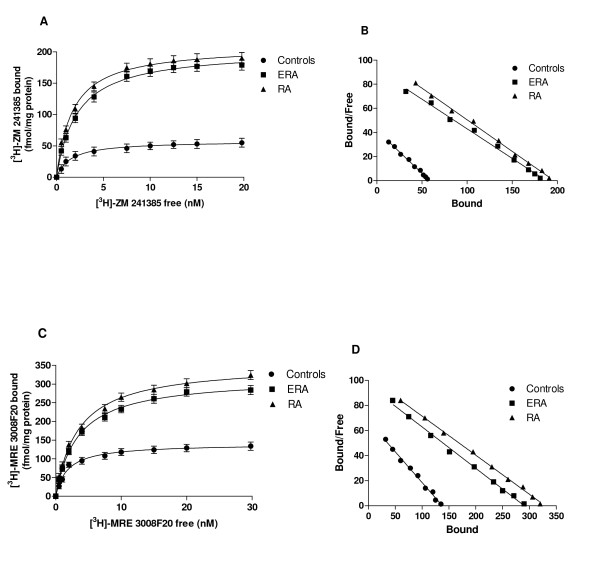
**Saturation binding experiments of A_2A _and A_3_ARs in RA patients**. Saturation curves (left) and Scatchard plots (right) showing the binding of (^3^H)-ZM 241385 to A_2A_ARs **(A **and **B) **as well as the binding of (^3^H)-MRE 3008F20 to A_3_ARs **(C **and **D) **in lymphocyte membranes derived from 90 healthy controls (•), 32 ERA patients (■), 63 RA patients (▲). Saturation binding experiments were performed as described in the online supplementary material and the data are reported in Table 2.

The affinity (K_D_, nM) and receptor density (B_max_, fmol/mg protein) of A_1_, A_2A_, A_2B _and A_3_ARs in lymphocyte membranes from healthy control group and RA patients are reported in Table 2. In lymphocyte membranes, the affinity and density of A_1 _and A_2B_ARs were not significantly different in RA patients (ERA and established), if compared with the control group. The affinity of the radioligands for A_2A _and A_3_ARs in ERA and RA patients was decreased (*, *P *< 0.01, Table 2) if compared with the control group. Moreover, A_2A _and A_3_AR density was significantly increased in ERA and RA patients compared with healthy subjects (Figure 2).

**Table 2:** Adenosine receptor binding parameters in lymphocyte membranes from healthy controls, ERA and RA patients

	(^3^H)-DPCPX A_1_ARs	(^3^H)-ZM 241385 A_2A_ARs	(^3^H)-MRE2029F20 A_2B_ARs	(^3^H)-MRE3008F20 A_3_ARs
**Healthy** **controls** ***n *= 90**	K_D _= 1.76 ± 0.08 nM Bmax = 34 ± 2 fmol/mg protein	K_D _= 1.41 ± 0.10 nM Bmax = 58 ± 4 fmol/mg protein	K_D _= 2.29 ± 0.14 nM Bmax = 55 ± 3 fmol/mg protein	K_D _= 1.92 ± 0.09 nM Bmax = 137 ± 9 fmol/mg protein
**ERA patients** ***n *= 32**	K_D _= 1.74 ± 0.06 nM Bmax = 33 ± 2 fmol/mg protein	K_D _= 2.09 ± 0.14* nM Bmax = 185 ± 12* fmol/mg protein	K_D _= 2.29 ± 0.10 nM Bmax = 55 ± 2 fmol/mg protein	K_D _= 3.03 ± 0.14* nM Bmax = 290 ± 15* fmol/mg protein
**RA** **patients** ***n *= 63**	K_D _= 1.69 ± 0.07 nM Bmax = 35 ± 2 fmol/mg protein	K_D _= 1.93 ± 0.12* nM Bmax = 195 ± 13* fmol/mg protein	K_D _= 2.36 ± 0.07 nM Bmax = 54 ± 2 fmol/mg protein	K_D _= 3.47 ± 0.21* nM Bmax = 328 ± 22* fmol/mg protein

ERA, early rheumatoid arthritis; K_D_, affinity constant, nM; Bmax, maximum density of specific binding sites, fmol/mg protein; RA, rheumatoid arthritis. Data are expressed as mean ± SEM. Differences were considered significant at a value of *P *< 0.01 vs healthy controls (*).

### A_2A _and A_3_AR agonists reduce NF-kB activation in RA

**Figure 3:**
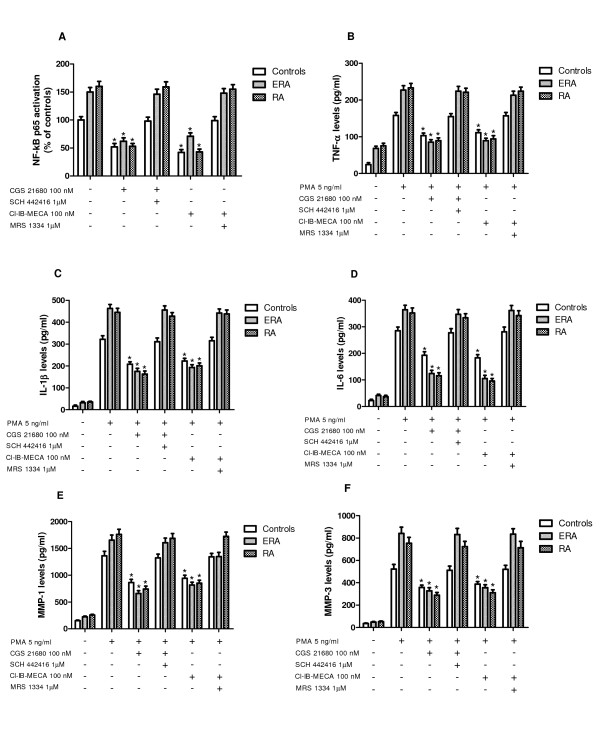
**Effect of A_2A _and A_3_AR stimulation in NF-kB, TNF-α, IL-1β, IL-6, MMP-1 and MMP-3**. Effect of a well-known A_2A_AR agonist and antagonist (CGS 21680, 100 nM; SCH 442416, 1 μM) or A_3_AR agonist and antagonist (Cl-IB-MECA, 100 nM; MRS 1334, 1 μM) in cultured lymphocytes of ERA (*n *= 30), RA patients (*n *= 30) and healthy subjects (*n *= 30) on: NF-kB activation **(A) **which was evaluated by detecting phosphorylated p65 proteins in nuclear extracts. The effect of the same compounds was also established in TNF-α release in control conditions and stimulated by PMA 5 ng/ml **(B) **and in IL-1β **(C) **and IL-6 levels **(D)**. The effect of the same compounds in monocytes from ERA, RA patients and healthy subjects in MMP-1 **(E) **and MMP-3 **(F) **activation was investigated. Functional experiments were carried out as described in the online supplementary material. Values are the mean and SEM. *, *P *< 0.01 versus controls (A); *, *P *< 0.01 versus PMA-treated cells (B-F).

Cultured lymphocytes of ERA and RA patients were characterized by high levels of activated NF-kB p65 in comparison with control subjects showing 1.5- and 1.6-fold increase, respectively (Figure 3A). CGS 21680 or Cl-IB-MECA (100 nM) were able to significantly inhibit NF-kB levels in the cultured lymphocytes derived from the subjects investigated. This effect was abolished by using well-known A_2A _and A_3_AR antagonists SCH 442416 and MRS 1334, respectively. The inhibitory effect mediated by A_2A _and A_3_AR agonists in RA patients was more than in healthy subjects (Figure 3A).

### Cytokine release is inhibited by A_2A _and A_3_AR stimulation in RA

The effect of A_2A _and A_3_AR agonists and/or antagonists on TNF-α (Figure 3B), IL-1β (Figure 3C) and IL-6 (Figure 3D) release was studied in lymphocytes. In cultured lymphocytes, without endogenous adenosine, obtained from ERA and RA patients, a marked release of TNF-α was observed, reaching 2.8- (ERA) and 3.1- (RA) fold of increase in respect to healthy subjects (Figure 3C). In addition, the stimulation of A_2A_AR with CGS 21680 mediated a significant inhibition of PMA-induced TNF-α release by 63% in ERA or by 62% in RA patients in comparison with control condition (35%). Similar results were obtained through the A_3_AR stimulation by using Cl-IB-MECA at the 100 nM concentration (60% in ERA, 57% in RA patients, 28% in healthy subjects). The inhibitory effect of A_2A _and A_3_AR agonists was counteracted by the antagonists SCH 442416 and MRS 1334 (1 μM), respectively.

As shown in Figure 3C, D basal levels of IL-1β and IL-6 released by cultured lymphocytes in RA patients were more than in controls. In addition, PMA (5 ng/ml) induced a marked release of these pro-inflammatory cytokines. The stimulation of A_2A _and A_3_ARs resulted in a significant reduction of IL-1β and IL-6 confirming their potential anti-inflammatory role in RA patients. The major effect was obtained in lymphocytes from RA patients most likely due to the up-regulation of A_2A _and A_3_ARs. A_2A_AR agonist mediated an inhibition of IL-1β and IL-6 in ERA (62% and 66%, respectively) or in RA patients (64% and 67%, respectively) in comparison with control subjects (Figure 3C). Similar results were obtained by using A_3_AR stimulation on IL-1β (Figure 3C) and on IL-6 release (Figure 3D). The direct involvement of A_2A _and A_3_ARs was demonstrated by using selective antagonists, SCH 442416 and MRS 1334, respectively, that were able to completely abrogate the inhibitory effect mediated by the agonists.

### A_2A _and A_3_ARs reduce MMP secretion in RA

Incubation of monocytes with PMA (5 ng/ml) for 24 hours induced MMP-1 and MMP-3 protein production. When monocytes were incubated with A_2A _or A_3_AR agonists the production of MMP-1 was inhibited in RA patients more than in healthy subjects (60% or 49% in ERA, 58% or 49% in RA patients, 37% or 29% in healthy subjects, respectively) (Figure 3E). Similar results were obtained evaluating the production of MMP-3 suggesting that these MMPs are closely associated with A_2A _or A_3_AR modulation (Figure 3F). The inhibitory effect of the A_2A _and A_3_AR agonists was blocked by the presence of selective antagonists demonstrating the direct involvement of these AR subtypes.

### A_2A _and A_3_AR binding parameters correlate with DAS28 and DAS

**Figure 4:**
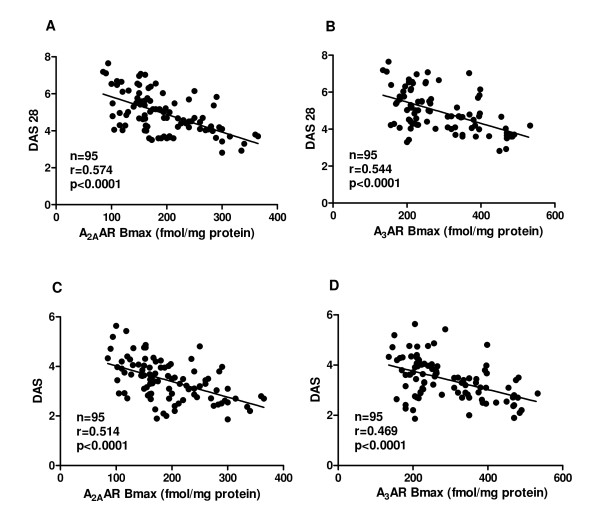
**Correlation between DAS28 or DAS and A_2A _or A_3_AR density**. Linear regression analysis between DAS28 or DAS and the receptor density expressed as Bmax (fmol/mg protein) of A_2A _**(A, C) **and A_3_ARs **(B, D) **in lymphocytes from 32 ERA and 63 RA patients considered as a whole.

An inverse correlation was found between DAS28 and A_2A _or A_3_AR density expressed as B_max _values in fmol/mg protein (Figure 4A, B). A similar correlation was also found between DAS and A_2A _or A_3_AR density (Figure 4C, D). No other significant correlation was found between A_2A _or A_3_AR affinity and DAS28 or DAS.

## Discussion

The primary aim of this study was to investigate the involvement of ARs in RA inflammatory responses and to assess potential relationships between these receptors and disease activity. In lymphocytes from healthy subjects (*n *= 90) ARs showed affinity values in the nanomolar range and a density from 34 to 137 fmol/mg protein. In ERA patients (*n *= 32) binding experiments have revealed a significant decrease of the affinity associated with an increase of A_2A _(3.2-fold) and A_3_AR (2.1-fold) density as demonstrated by Bmax values (Table 2). Similarly in established RA patients (*n *= 63) an up-regulation of A_2A _and A_3_ARs density values was found. mRNA assays and Western blotting with densitometric analysis supported that A_2A _and A_3_ARs are higher in RA patients in respect to control subjects. These data confirmed our previous results obtained in a small cohort of ERA (*n *= 20) and RA patients (*n *= 23) showing an increased A_2A _and A_3_AR density compared with healthy (*n *= 30) individuals [30]. In that study our group found that in RA patients treated with anti-TNF-α agents, A_2A _and A_3_AR density was similar to control subjects demonstrating an active role of TNF-α in the up-regulation of these receptors, as was also suggested by literature data [38-40]. To date, it has been reported that A_3_ARs in peripheral blood mononuclear cells of patients with autoimmune inflammatory diseases, such as RA, psoriasis and Crohn's disease, are over-expressed if compared with healthy subjects [27]. A highly specific orally bioavailable A_3_AR agonist, CF101 has been used in active RA patients where A_3_ARs are overexpressed in synovial tissue and in peripheral blood mononuclear cells [29].

In this report, we verified whether the increase in A_2A _and A_3_AR density was associated with inflammatory cellular responses, such as NF-kB, TNF-α, IL-1β, IL-6, MMP-1 and MMP-3. The ability of CGS 21680 or Cl-IB-MECA to inhibit NF-kB activation was studied showing an increase of their effect in ERA and RA patients. Currently, there is considerable interest in developing specific alternative pharmacological treatments reducing NF-kB activation and pro-inflammatory cytokine production in RA through the involvement of A_2A _and A_3_ARs [16]. It has been reported that A_3_AR overexpression in RA patients induced by inflammatory cytokines is able to reduce NF-kB activation [41]. The complex studies performed on NF-kB activation clearly demonstrated a central role for p65 or p50/p65 heterodimers in the inflammation that underlies RA [13]. It is well known that NF-kB positively regulates gene encoding cytokines, such as TNF-α, IL-1β, IL-6 and other inflammatory factors, suggesting that this transcription factor could be one of the master regulators of inflammatory cytokine production in RA [42]. Animal models of inflammatory arthritis also support the concept that NF-kB activation happened prior to the onset of clinical manifestations of arthritis [43]. In a murine type II collagen-induced arthritis, it was found that NF-kB expression correlated with MMP-13 and MMP-3 levels leading to cartilage destruction and articular damage [44].

It was also reported that several cytokines may participate in the pathogenesis of cartilage damage and TNF-α represents one of the principal cytokines linked to the cartilage destruction. In our experimental conditions the stimulation of A_2A _and A_3_ARs reduced the high levels of TNF-α found in ERA and in RA patients, probably via an inhibition of NF-kB. These data are in agreement with previous results showing that the activation of A_2A _and A_3_ARs suppresses proinflammatory cytokines [25,45]. It is well known that different cytokines are expressed and are functionally active in the synovial tissue or in peripheral blood cells of RA patients [11,46]. We found that the stimulation of A_2A _and A_3_ARs mediated a significant decrease of IL-1β or IL-6 release in RA patients in comparison with healthy subjects. These data suggest a direct involvement of adenosine in the expression of IL-6 that represents a key pro-inflammatory cytokine associated with the severity of the joint damage and osteoclastogenesis [12,47,48].

The destruction of articular cartilage is a typical pathologic feature of arthritic diseases such as RA mediated by proteases belonging to MMP class enzymes [43]. Among these, MMP-1 and MMP-3 are considered to be of particular interest since they directly degrade the components of the cartilage matrix, including aggrecan and collagen [13]. Proinflammatory cytokines, such as TNF-α and IL-1β, stimulate the production of MMPs through the activation of cellular signaling pathways involving NF-kB [42,49]. We have investigated the effect of A_2A _and A_3_AR stimulation on MMP-1 and MMP-3 production showing a significant inhibition in ERA and RA patients in respect to healthy subjects. In RA patients, the increase of A_2A _and A_3_AR density reflected an increase in receptor functionality suggesting a role of adenosine in the reduction of inflammatory status and in cartilage degradation induced by MMP activity and expression. These data are in agreement with those found in studying the inhibitory effect of AR stimulation on MMP-1 and MMP-3 expression in synovial fibroblasts [15,50].

The progression of RA is quite heterogeneous, ranging from very mild to rapidly progressive and debilitating forms. To optimize the management of the disease, an international task force of rheumatologists has recently delivered recommendations based on the new therapeutic paradigm known as "treat to target". In this new scenario the close monitoring of DAS28 or DAS aimed to reach a state of remission, or alternatively a state of low disease activity, is used by an attending clinician to adjust pharmacological therapy [51]. At the present time due to the multifaceted nature of RA, no single clinical or laboratory parameter is able to describe satisfactorily the level of inflammatory activity or the disease prognosis at any given time [8]. Recently, it has been reported that the presence of a relationship between DAS28 and serum adenosine deaminase (ADA) levels suggests that ADA concentration may predict disease activity in RA patients [52]. Our data, for the first time, show an inverse correlation between disease activity measured by DAS28 or DAS and A_2A _or A_3_AR density confirming a relationship between the inflammation and adenosine in RA.

## Conclusions

This paper suggests a correlation of A_2A _and A_3_AR with the inflammatory and clinical responses in RA and that their up-regulation could represent a compensatory mechanism to better counteract the inflammatory status. In particular, the highest levels of A_2A _and A_3_AR density are closely associated with the lowest levels of DAS28 and DAS, suggesting that the endogenous activation of these receptors attenuates the disease activity. From the pharmacological point of view, it could be of crucial importance that the stimulation of the over-expressed A_2A _and A_3 _ARs leads to the inhibition of cellular pro-inflammatory and degenerative mediators.

The novel findings of this study are represented by a high inverse correlation between A_2A _or A_3_AR density and the DAS28 or DAS validated index of disease activity in RA. Moreover, our results suggest the use of A_2A _and/or A_3_AR agonists as novel potential pharmacological treatment combined with the classical therapy in human diseases characterized by a marked inflammatory component as in RA.

## Abbreviations

ACR: American College of Rheumatology; ADA: adenosine deaminase; ANOVA: analysis of variance; anti-CCP: anti-cyclic citrullinated peptide; ARs: adenosine receptors; CGS 21680: 4-(2-((6-Amino-9-(*N*-ethyl-β-D-ribofuran uronamidosyl)-9*H*-purin-2-yl)amino)ethyl)benzene propanoic; Cl-IB-MECA: N^6^-(3-iodo-benzyl)-2-chloro-adenosine-5'-*N*-methyluronamide; DAS: disease activity score; DMARDs: disease modifying anti-rheumatic drugs; DPCPX: 1,3-dipropyl-8-cyclopentyl-xanthine; ELISA: enzyme linked immunosorbent assay; ERA: early rheumatoid arthritis; HAQ: Health Assessment Questionnaire; HRP: horseradish peroxidase; IL-1β: interleukin-1β; IL-6: interleukin-6; MMP-1: matrix metalloproteinase-1; MMP-3: metalloproteinase-3; MRE 2029F20: N-benzo(1,3)dioxol-5-yl-2-(5-(2,6-dioxo-1,3-dipropyl-2,3,6,7-tetrahydro-1*H*-purin-8-yl)-1-methy-1*H*-pyrazol-3-yl-oxy)-acetamide; MRE 3008F20: 5N-(4-methoxyphenylcarbamoyl)amino-8-propyl-2-(2-furyl)pirazolo (4,3-e)-1,2,4-triazolo(1,5-c)pyrimidine; MRS 1334: 1,4-Dihydro-2-methyl-6-phenyl-4-(phenylethynyl)-3,5-pyridinedicarboxylic acid 3-ethyl-5-((3-nitrophenyl)methyl) ester; NF-kB: nuclear factor kB; PMA: phorbol myristate acetate; RA: rheumatoid arthritis; RF: rheumatoid factor; RT-PCR: real time quantitative polymerase chain reaction; SCH 442416: 2-(2-Furanyl)-7-(3-(4-methoxyphenyl)propyl)-7*H*-pyrazolo(4,3-*e*)(1,2,4)triazolo(1,5-*c*)pyrimidin-5-amine; SEM: standard error of the mean; TNF-α: tumor necrosis factor-α; ZM 241385: 4-(2-(7-amino-2-(2-furyl)(1,2,4)-*triazolo*(2,3-a)(1,3,5)triazin-5-ylamino) ethyl) phenol

## Competing interests

The authors declare that they have no competing interests.

## Authors' contributions

All authors were involved in drafting the article or revising it critically for important intellectual content, and all authors approved the final version to be published. PAB had full access to all of the data in the study and takes responsibility for the integrity of the data and the accuracy of the data analysis. KV, MG and PAB participated to the study conception and design. KV, FV, MT and MP were responsible for the acquisition of data. The analysis and interpretation of data were performed by KV, MG, FT and PAB. All authors read and approved the final manuscript.
